# A Comprehensive Review of Lesion Sterilization and Tissue Repair: An Alternative for Pulpectomy in Deciduous Teeth

**DOI:** 10.7759/cureus.48218

**Published:** 2023-11-03

**Authors:** Aparna Achanta, Amit Reche, Rishika Dakhale, Rudra R Bharate

**Affiliations:** 1 Public Health Dentistry, Sharad Pawar Dental College and Hospital, Datta Meghe Institute of Higher Education and Research, Wardha, IND

**Keywords:** conservative management, pediatric dentistry, dental caries, non-invasive technique, triple antibiotic paste

## Abstract

Pulpectomy has always been a treatment choice when irreversible damage to the pulpal and periapical tissues occurs, but in a pediatric patient, expecting cooperation and adequate chair side time is a huge task as they fear the dentists and the instruments. Hence, it was required that there come an advanced treatment protocol that helps overcome the drawbacks of the existing treatment modality. A new therapeutic strategy called lesion sterilization and tissue repair (LSTR) is revolutionizing how dental lesions are treated today. Through a non-invasive antimicrobial treatment, this cutting-edge method seeks to halt the spread of carious lesions while encouraging tissue repair and regeneration. In LSTR, the infected tooth tissues are treated directly with a special antimicrobial combination of antibiotics, reducing agents, and disinfectants. Through the elimination of the microorganisms that cause dental decay and the creation of an environment that is favorable for natural tissue healing, this intervention targets the microbial etiology. This article mainly focuses on using antibiotics as a treatment modality in a tooth affected by caries and where the pulp and periradicular tissues are irreversibly inflamed. Pulpectomy has always been the most practiced technique, but in children, due to the lack of cooperation during the treatment and harm to the underlying developing tooth bud, a less invasive protocol had to be used, which is LSTR. This review delves into the basic ideas, techniques, and clinical applications of lesion sterilization and tissue restoration to look at its various aspects. This review clarifies the effectiveness and safety of LSTR in controlling diverse dental diseases, ranging from early-stage caries to deep dentin infections, by critically assessing previous study findings. It also emphasizes LSTR's ability to preserve vital tooth pulp, obviating the need for invasive and frequently uncomfortable endodontic operations. The review advocates for integrating LSTR into contemporary dental practices, showcasing its potential to transform the landscape of oral healthcare. By providing a comprehensive overview of this innovative technique, this review encourages further research and clinical implementation, heralding a new era in dentistry focused on preservation, regeneration, and enhanced patient well-being.

## Introduction and background

In a regular dental practice for pediatric patients, the treatment options for deep dental caries are limited, especially in cases where the surrounding periodontal tissue is also affected; the classical approach is generally tooth extraction followed by delivering space maintainers or regainers if indicated [1]. It is a well-known fact and mentioned in the literature that a natural tooth is the best space maintainer in mixed and developing deciduous dentition; thus, every possible attempt should be made to preserve it. Moreover, delivering a space maintainer or space regainers requires a lot of cooperation from the patient. It will become tough for young patients who do not cooperate with the treatment [1-2]. There came a need for lesion sterilization and tissue repair (LSTR), a safer and less invasive method of management of deep carious lesions. A pulpectomy is not an indicated option due to the root resorption or risk of injury to the underlying developing tooth bud. When a tooth is extracted in a developing dentition stage, the space that has been created has to be preserved, as early tooth loss can cause problems such as aberrant eruption, altered eruption sequence, and space loss emergence of undesirable habits and can compromise speech and function. The development of occlusion depends heavily on the growth of primary teeth [2-3].

The most promising space maintainer is a primary tooth successfully treated and fixed. Primary teeth tend to have meandering root canals, ramifications, numerous accessory canals, and large medullary bone gaps, all promoting the spread of infection. Due to physiologic root resorption of the primary tooth, there is no apical closure, making it difficult to achieve a hermetic seal in the primary teeth [3]. The proximity of the developing permanent tooth germ to the roots of the primary teeth is another obstacle in endodontic therapy. The behavior management of resistant children is an additional challenge for the dentist in providing efficient endodontic therapy [4]. However, due to the complex structure of root canals, instrumentation alone cannot completely eradicate pathogens from the pulp chamber system [5].

Consequently, one approach to eliminating bacteria in the root canal procedure is the local use of antibacterial medications. It is doubtful that a single antibiotic could result in the efficient and reliable disinfection of all canals due to the intricacy of root canal infections dealing with the varied flora encountered; a combination would probably be required. Combining antibiotics would also lessen the possibility of bacterial strains becoming resistant [5]. Tooth decay is a common oral health problem, particularly in youngsters. It needs efficient treatment approaches that put the patient's requirements and the preservation of healthy tissue first. For carious lesions in deciduous teeth, a method called LSTR is now available. Using a mix of antibacterial medications, LSTR therapy allows for the disinfection of dentinal, pulpal, and periradicular lesions [6]. The LSTR concept was developed at the Cariology Research Unit, School of Dentistry, Niigata University, Japan, 2004 [7]. LSTR is an emerging therapeutic approach that revolutionizes the conventional methods of treating dental lesions. This innovative technique aims to arrest the progression of carious lesions through a non-invasive antimicrobial strategy, promoting tissue repair and regeneration. LSTR involves the application of a unique antimicrobial mix comprising antibiotics, reducing agents, and disinfectants directly onto the affected dental tissues. This intervention targets the microbial etiology, eliminating pathogens responsible for dental decay while creating a conducive environment for natural tissue healing. This review aims to explore the history, principles, and clinical applications of LSTR, shedding light on its potential as a valuable tool in pediatric dentistry. This review aims to explore the history, principles, and clinical applications of LSTR, shedding light on its potential as a valuable tool in pediatric dentistry [5-7].

## Review

Search methodology

**Figure 1:**
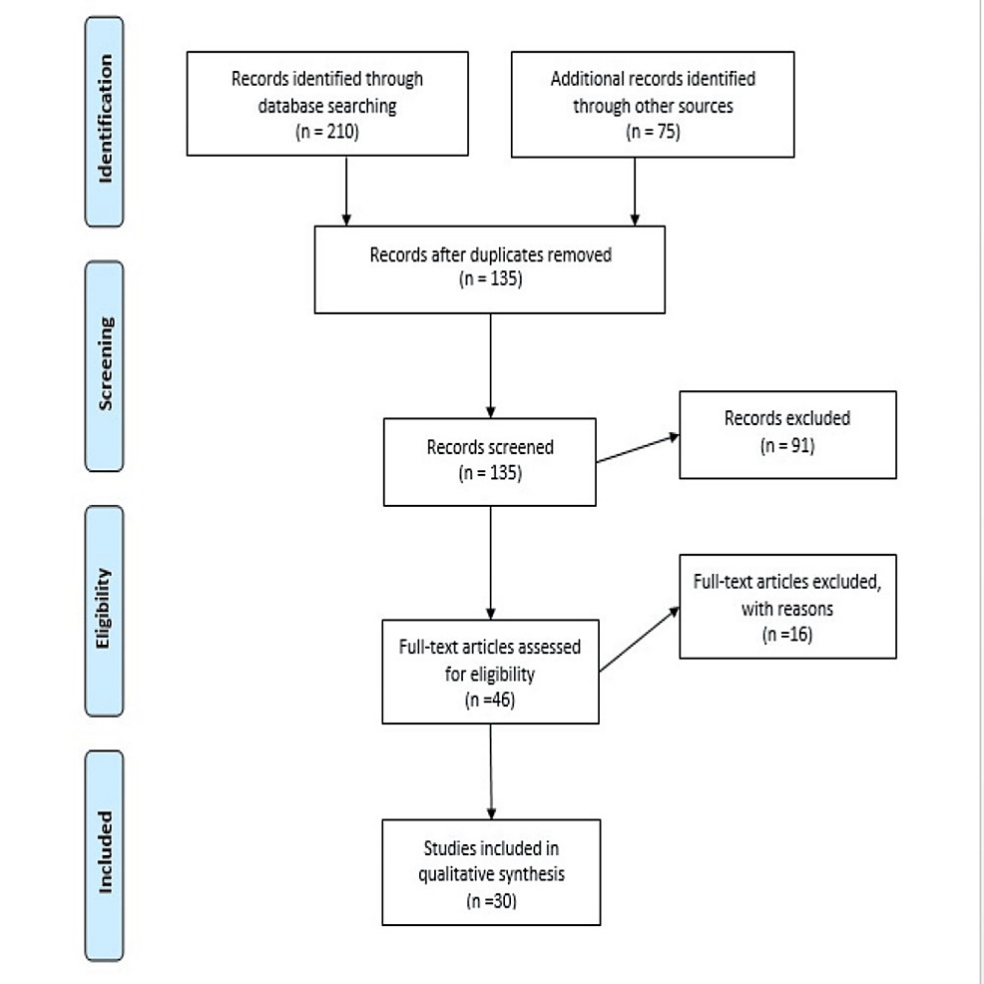
PRISMA flow diagram for search strategy. Adopted from PRISMA PRISMA, Preferred Reporting Items for Systematic Reviews and Meta-Analyses

A comprehensive search was made to gather literature on LSTR utilizing electronic databases, such as PubMed, Scopus, and Web of Science. Keywords such as “noninvasive,” “minimally invasive,” “conservative management,” “dental caries,” “pediatric dentistry,” and “triple antibiotic paste” were used to search the database. Articles focusing on LSTR were included, but publications focusing chiefly on pulpectomy and extractions were omitted. The inclusion criteria included relevant books, articles, studies, conference presentations, gray literature or unpublished literature, and reviews. The study selection procedure had screening titles and abstracts, followed by a full-text evaluation of relevant papers. The final group of included research offers a thorough analysis of the evidence that is currently available on the uses of LSTR. The results were combined and analyzed to draw meaningful conclusions. Figure 1 describes the selection process of articles used in our study.

Concept of LSTR

The LSTR concept was developed at the Cariology Research Unit, School of Dentistry, Niigata University, Japan, 2004 [7]. It is a concept that aims at the preservation of natural tooth structure to the extent possible, instead of the standard treatment, which includes the extraction of teeth. If lesions are disinfected, injured tissues will likely heal. A single antibiotic is unlikely to disinfect a root canal due to the polymicrobial nature of an infected root canal. Hence, a combination of antibacterial drugs is used [8]. There is a high chance that, even after the pulpectomy and endodontic instrumentation in a deciduous tooth, the infection may persist because of the tortuous root canal morphology and the natural root resorption. Due to inadequate apical seal, there is always a leak of the microorganism from the apical end of the tooth, and this infection may ultimately spread to the surrounding periodontal tissues and even the developing tooth bud. LSTRs are treatment modalities where, with the least possible instrumentation or even no filing or tooth preparation, the complete disinfection of the tooth can be achieved by placing a mixture of two or three antibiotics [8]. The selection of antibacterial drugs has been done based on various studies related to bacterial isolation from oral sites, including endodontic lesions of primary teeth [8]. A preparation of ciprofloxacin metronidazole and minocycline in a 1:3:3 ratio, famously called the triple antibiotic paste (TAP), is used for LSTR. The use of this combination shows effective results in combating the microorganisms, helps in the regeneration of the secondary dentin, and even stimulates the healing of the periodontal ligament.

TAP

The first use of antibiotics in endodontic procedures is dated a long way back in 1951 when Grossman made use of a poly-antibiotic formula, which is a combination of penicillin, bacitracin, streptomycin, and caprylate sodium (PBSC), which was highly effective back then. Even though PBSC's clinical examination demonstrated positive therapeutic effects, the formula unaffected anaerobic bacteria, which are crucial to endodontic problems. As a result, the United States Food and Drug Administration outlawed the use of PBSC for endodontic procedures in 1975, citing the possibility of hypersensitivity and penicillin allergy. TAP is an antibiotic combination that was created specifically to aid in the process of rejuvenation. Divya et al. introduced it after studying its efficiency in eliminating germs from the root canal. This mixture is used to treat necrotic pulp in open apex teeth because it is a powerful anti-microbial agent with various applications in endodontics [9]. TAP, a strong antibacterial agent, has several applications and is employed in various ways. By attempting to regenerate and revascularize the diseased pulp, it is utilized to preserve the pulp's life. In addition to being a powerful disinfectant, TAP is essential for regeneration and revascularization [10].

Preparation of TAP

Malu et al. in 2022 used a ratio of 1:1:1 (metronidazole 500 mg, ciprofloxacin 200 mg, minocycline 100 mg) in making the combination of antibiotics [11]. Takushige et al. 1998 modified the above ratio to 1:3:3 of the same antibiotics mentioned above [12]. All the antibiotics are powdered and mixed on a sterile paper pad or mortar pestle with an appropriate solvent to form a paste. The formed mixture is opaque in color and should be stored in an airtight container, and care must be taken that, if this mixture turns translucent, it should be discarded. The TAP can be prepared in several other combinations to overcome the drawbacks of the components and prepare a better mix. Sato et al. [6] suggested replacing minocycline with other antibiotics such as amoxicillin and fosfomycin, which are as effective as the previous component. This change was suggested as minocycline caused darkening of the tooth structure [12]. Burrus et al. suggested using clindamycin instead of minocycline as clindamycin is more effective against anaerobic microorganisms and streptococcal strains of bacteria [4].

Factors influencing the action of TAP

The primary factor determining the success of the treatment is the amount of drug being used since the drug should be sufficient for it to diffuse in a downward direction toward the apex into the tissue surrounding the tooth's root [12]. Pulp and other periradicular tissue infections are generally caused by multiple aerobic and anaerobic microorganisms; hence, more than one antibiotic with broad-spectrum activity must be used. It should be ensured that the drug being used is biocompatible and does not harm the host cells of the tissue while possessing antimicrobial activity. The smear layer formed during the cavity preparation must be cleaned thoroughly as it will inhibit the diffusion of the drug. This removal of the smear layer can be done using chemical agents such as ethylenediaminetetraacetic acid (EDTA) or can be done using ultrasonic equipment; this will help in opening the dentinal tubules, thus allowing for better penetration of the drug into the tooth [13].

Procedure of LSTR

The very first step is the preparation of the TAP. The paste can either be freshly prepared right before the procedure or prepared well before and stored in an air-tight container away from sunlight and moisture. After this step, the local anesthetic agent (LA) is administered, and the additional application of a topical local anesthetic helps reduce the pain and discomfort. Once the action of LA sets in, the rubber dam is placed to maintain thorough isolation throughout the procedure. After this, the access cavity is prepared using a round bur, so any necrotic pulp tissue present coronally is wholly removed. Any existing previous restoration and the caries also have to be removed. Irrigation is then performed using saline and sodium hypochlorite. EDTA is a very effective agent that can be used since it eliminates the smear layer, allowing for clean, clear dentinal tubules that enable antibiotics to penetrate more deeply. If bleeding occurs, sodium hypochlorite, a powerful hemostatic substance, can stop. It prevents the pulpal hemorrhage that jeopardizes pulpal healing, is nontoxic to pulpal tissue, does not interfere with pulpal healing, and clots can be removed. After thoroughly rinsing and drying the prepared access cavity, the medication cavity is generally prepared at the orifice of the canal opening. The cavity is prepared using a round bur approximately 2 mm deep and 1 mm wide. This medication cavity holds the TAP in place, allowing its action to occur. The paste is inserted into the cavity and allowed to properly cure before being permanently restored using glass ionomer cement and a stainless steel crown. Postoperative follow-up should be done, and the reduction in earlier clinical symptoms, such as pain and tenderness, should be recorded. The preoperative and postoperative radiographs should be compared for the changes in periapical and furcation radiolucency and infection if any [1-4].

Indications of LSTR

The primary indication of LSTR is the patient's complaint of pain and discomfort. In a primary tooth, the A-delta fibers are less developed. Hence, the initial or reversible pain is not experienced by the child directly feel the pain when it intensifies when the C fibers are stimulated; in such cases, the pulp gets irreversibly involved and has to be extirpated, and in these selective cases, LSTR can be performed [11]. A tooth with root resorption, such as pulpectomy and instrumentation, may injure the developing tooth bud [14]. A tooth involving the periodontal ligament, with a significant widening of the lamina dura, is identifiable in a radiograph [7]. The presence of a draining sinus and periapical abscess in the affected tooth is an absolute indication for the procedure of LSTR as this draining sinus is indicative of an infection that has to be treated with antibiotics [14].

Contraindications of LSTR

An absolute contraindication of LSTR is the history of sensitivity or allergy to any of the antibiotics used in the preparation of the TAP [15]. Patients with a history of infective endocarditis cannot be treated with LSTR [15]. A tooth with excessive root resorption and physiologic mobility cannot be indicated for LSTR. A tooth with perforation in the pulpal floor and a destructed crown such that a proper restoration or seal post-treatment cannot be achieved cannot be treated with LSTR; as in cases of the perforation of the pulpal floor, there is a fear of the antibiotics leaking beyond the tooth into the periapical and periradicular space. As for the tooth with a destructed crown, the post-restorative prosthesis cannot be placed, which is a necessary component of the procedure to prevent microleakage and further destruction of the crown. Hence, LSTR is contraindicated [16].

Advantages of LSTR

It is a cost-friendly and effective treatment modality that can be used for all primary teeth [17]. The tooth that does not provide a favorable area for instrumentation can be easily treated with this technique as this is less invasive, and practically no instrumentation is required for this technique. Retreatment, if necessary, can be easily performed as no obturation or sealing of the root canals is performed [18]. This mode of treatment leads to less chair-side time for the patient, thus enhancing better cooperation. This treatment is economical and easier as compared to pulpectomy.

Disadvantages of LSTR

The use of antibiotics for the treatment has raised multiple questions over time as there are several side effects of the drugs that are used as they may even diffuse systemically; the use of these for a local purpose is not recommendable [19-20]. The TAP is radiolucent, and, hence, radiographically, the evidence of either the success or failure of the treatment cannot be analyzed [4]. The sensitivity to antibiotics, development of resistant microbial strains, the risk of harm to the developing permanent tooth bud, and allergy to the antibiotics add up to the disadvantages of this treatment [19].

Discussion

Dental caries are among the most common oral diseases, particularly among youngsters. Although typical restorative therapies have effectively managed carious lesions, they have the potential to result in a large amount of tooth structure being removed, which raises the risk of pulpal exposure and subsequent problems. Targeting the bacterial infection within the carious lesion, letting the afflicted tooth recover naturally, and minimizing the need for invasive restoration operations is how LSTR addresses this concern [20]. In children, the nerve fibers responsible for early pain stimulation are not very well-developed; hence, the reversible condition is not identified unless observed by a dentist clinically. The common happening, thus, is that the patient comes in with severe pain such that the tooth has to be treated by either extraction or pulpectomy [20]. LSTR has been used recently as a treatment modality in such cases. The main aim of treatment is the complete removal of diseased tooth structure coronally and the removal of infection, which is in the root canal space, and any infection that has spread beyond the apex [11]. The TAP used for this treatment works very well to remove any infection and the bacterial toxins that have been created. Using three antibiotics, metronidazole, ciprofloxacin, and minocycline, in a ratio of 1:3:3, helps provide a broad spectrum activity, thus ensuring action on both the aerobic and anaerobic microorganisms. This combination of drugs can be modified by replacing any of the components with a drug producing a similar action according to the availability and depending on the patient's sensitivity. LSTR is a noninvasive technique, ensuring no harm and least or nil instrumentation, thus reducing the in-chair time of the child and enhancing the cooperative behavior, thus providing a quality treatment [1]. Takushige et al. [1] performed LSTR in around 87 clinical cases, out of which 70 cases, which accounts for up to 70% of the cases, showed a successful result with a reduction in the symptoms, and the post-treatment radiographs showed a reduction in the periapical and furcation involvement radiolucency. Takushige et al. prepared an access opening and then removed the necrotic tissue and the existing previous restoration, if any; cleaned the walls of the cavity with 35% phosphoric acid; and prepared a medication cavity of around 3 mm in the orifice of the root canal to place the TAP [1]. No preparation was done in the root canal space. Hence, this technique got its name for being minimal or noninvasive [1]. This minimally intrusive strategy encourages the body's inherent healing mechanisms, enabling tissue regeneration and repair while concentrating on eliminating the infection. The use of LSTR procedures emphasizes the value of individualized, patient-focused dental care. In the long run, this preserves the integrity of the dentition by minimizing the influence on the healthy tooth structure as well as the discomfort that patients experience. LSTR is mainly based on the use of a combination of antibiotics, varied combinations have been used, and many more studies are being done to come up with a drug combination beneficial in all aspects [21].

Prabhakar et al. performed a study advocating that the LSTR treatment gives a better prognosis in a primary tooth by eliminating the bacteria, reducing the infection, and promoting regeneration [22]. Verma et al. in their randomized control trial mentioned the use of clindamycin-based TAP, which ensured a good result compared to the one based on minocycline [23]. Many researchers till date have studied various treatment modalities, and they agree that complete removal of the pulp organ is not a feasible technique, so the use of any such material, which will help in the proper healing of the tissues and give a good prognosis, can be utilized and the use of TAP for LSTR is one such option, which is now being used in further studies by several practitioners and researchers [24-25]. While preparing the canals using hand files or even an endomotor, it ought to happen that the preparation is not sufficient to eradicate the complete necrotic tissue, which is present in the tooth. Studies are suggestive of the fact that nearly 50% of the canal may remain uninstrumented. This may lead to the regrowth of any residual microflora as they may derive their nutrition from the remaining necrotic debris [26-28]. Removal of the causative microorganisms from the infected site helps in better healing and regeneration of the lost tissues can be achieved by doing so as well. LSTR does this work very efficiently as this technique is based on the application of antibiotics locally to curb the infection and reduce inflammation. Clinical trials and research studies have demonstrated promising results with the use of LSTR. It has shown effectiveness in halting the progression of carious lesions, providing an alternative to invasive treatments such as pulp therapy or extractions. The continuous evolution of LSTR demands further research to establish optimal protocols and long-term outcomes. Collaborative efforts between researchers and clinicians are essential to improve the evidence base and establish LSTR as a reliable management option for carious lesions in primary teeth [29-30].

## Conclusions

In conclusion, LSTR offers ground-breaking treatments for dental caries and other oral lesions, representing a paradigm change in dentistry. Dental professionals can deliver more effective and minimally intrusive interventions by customizing treatments to each patient's needs and considering the body's intrinsic capacity for healing. Pulpectomy, on the other hand, though practiced for ages, has several drawbacks that can be overcome by LSTR. LSTR also exemplifies the growing trend in contemporary medicine toward holistic and preventive therapies. Dental experts can significantly improve general health and well-being by addressing dental problems at their physical and symbolic roots. Given the complicated relationship between dental health and various medical disorders, the emphasis on tissue repair ensures oral health restoration and promotes systemic health. LSTR illustrates the proactive spirit of dentistry research by demonstrating the sector's dedication to discovering innovative methods to benefit patients. These methods can reset the standards for oral healthcare, making procedures more efficient and patient-friendly as they develop and become more widely used in dental practices. Ultimately, LSTR represents a significant step toward a future where dental therapies actively promote the body's natural healing systems, creating healthier smiles and, consequently, healthier lives rather than simply treating the symptoms.
